# CHILDHOOD PARENTAL RELATIONSHIP AND COGNITIVE HEALTH IN LATER LIFE: ASSOCIATIONS MEDIATED BY SELF-ESTEEM AND SENSE OF CONTROL

**DOI:** 10.1093/geroni/igae098.0788

**Published:** 2024-12-31

**Authors:** Xi Zhu

**Affiliations:** Baylor University, Waco, Texas, United States

## Abstract

Life course researchers have found that early-life experiences are linked to later-life cognitive health. However, very few studies have paid specific attention to the parent-child relationship in childhood. This study extends the current literature by considering the relationship between childhood parental relationship and cognitive health in later life. Using data from the National Longitudinal Study of Youth (1979-2020), I looked at both positive and negative/dysfunctional parental relationships in childhood and examined whether they predicted older adults’ self-rated memory and objective cognitive function. To further understand the mechanisms behind the relationship, I also examined the mediation role of self-esteem and sense of control. Findings show that childhood parental love and affection are related to better self-rated memory in later life. Dysfunctional parental relationship is associated with worse self-rated memory. Childhood parental relationships show null association with objective cognitive function among older adults. In addition, KHB mediation tests suggest that self-esteem and sense of control significantly mediated the relationship between childhood parental relationships and later-life subjective cognition. This study contributes to a deeper understanding of the complex factors that influence cognitive health and self-perception of memory in older adults. It suggests that parent-child relationship from early life has a long-term impact on how individuals perceive their cognitive abilities. It also indicates that enhancing psychological resources might have a positive effect on self-perceived cognitive health in older adults. Furthermore, this study highlights the need for clinicians and researchers to distinguish between subjective self-assessments and objective cognitive tests in evaluating older adults’ cognitive health.

